# Provisional Matrix Formation at Implant Surfaces—The Bridging Role of Calcium Ions

**DOI:** 10.3390/cells11193048

**Published:** 2022-09-29

**Authors:** Eduardo Anitua, Ricardo Tejero

**Affiliations:** 1 Private Practice in Oral Implantology, 01007 Vitoria-Gasteiz, Spain; 2 BTI Biotechnology Institute I+D, 01510 Vitoria-Gasteiz, Spain; 3 University Institute for Regenerative Medicine and Oral Implantology (UPV/EHU), 01007 Vitoria-Gasteiz, Spain

**Keywords:** implants, surface engineering, surface chemistry, coagulation, osseointegration, adsorption, implant surgery, sinus lift, calcium ions

## Abstract

The success of dental implants lies in their strong and lasting integration into the patient’s receiving bone. The first biological interactions at the implant surface determine the subsequent evolution of the integration process. In this study we set our objective to analyze the mechanistic interaction of the early regenerative matrix at implant surfaces modified with calcium ions (Ca) as compared to standard implant surfaces (NoCa). We put the surfaces in a Quartz Crystal Microbalance with Dissipation (QCM-D) to monitor the frequency shift (*f*) and the viscoelastic properties of the adsorbed biofilms and used Scanning Electron Microscopy (SEM) to visualize the resulting interfaces. Upon the addition of human blood plasma, Ca surfaces formed an adsorbed three-dimensional film attached to the surface (∆*f* = −40 Hz), while with NoCa, the biofilm formed but was not attached to the surface (∆*f =* 0 Hz). After 20 min in blood, two representative commercial implants with Ca and NoCa surfaces showed also distinct interfaces: Ca implants formed a visible clot attached to the implant which was composed mainly of platelets (Surface Coverage: 40 ± 20%) and some red blood cells (SC: 9 ± 3%) entrapped within a fibrin network (SC: 93 ± 5%). The NoCa implants were largely populated by red blood cells (SC: 67 ± 12%) with scarce fibrin remnants (SC: 3 ± 2%), and the implants showed no clot on their surfaces macroscopically. The pre-clinical and clinical results discussed in this work encourage the modification of titanium implant surfaces with calcium ions to improve the bone regenerative process. Taken together, these results add more information about the roles of Ca ions in bridging the formation of the provisional matrix at implant surfaces and their effects on implant osseointegration.

## 1. Introduction

Dental implantology has become a common practice for the restoration of the aesthetics and masticatory functions of millions of people every year. Restorative implant dentistry relies on the long-term integration of the implanted material in bone. This process is modulated by surgical factors such as the surgery plan and the prosthetic loading strategy; biological factors such as the patient’s health and the receiving bone conditions; and implant-related factors such as the base material characteristics, the surface physiochemistry, topography, final quality, and cleanliness of the manufactured implant [1,2]. Over the last decades, the evolution of implant designs, prosthetic constructions, and surface characteristics have facilitated surgeries and improved patient satisfaction in increasingly challenging situations [3,4].

Peri-implant bone regeneration begins with the placement of an implant and the quasi-instantaneous establishment of a new ionic balance between the surface and the receiving bed. In the quest for faster and better bone-tissue integration, implant surface designs have historically sought to imitate the structure and composition of bone tissue. Long story short: a bone-like structure at the implant surface should more easily fuse with the existing bone bed. Typically, these modifications consisted of calcium phosphates with or without collagen or other non-collagenous proteins with key roles in regeneration such as osteopontin and osteocalcin, which aimed at mimicking, as much as possible, the inorganic and organic part of the mature bone matrix of the later stages of bone regeneration. However, these solutions faced several limitations that have undermined their final clinical relevance [2].

An alternative strategy consists of focusing on the early phases of implant–bone tissue interaction. Immediately upon implant insertion, the establishment of an ionic equilibrium at the implant–tissue interface is determined by the chemical composition of the implant surface and by the local ionic composition. The surface adsorption and exchange of proteins with the biological environment, which is a fundamental part of the process of hemostasis and inflammation [5,6,7], follows. The ionic configuration of the implant surface is therefore key to the subsequent responses down the line [8]. Importantly, ionic modifications allow a more straightforward application on an industrial basis, overcoming the limitations of the more complex bone-like coatings [3,5,6,9,10]. 

In 1992, Ellingsen et al. postulated that calcium ions adsorbed from blood upon implantation onto the titanium oxide surface were initiators of the process of osseointegration at implant surfaces. The double-charged positive calcium ion would serve as an electrostatic bridge to bring together the negatively charged titanium oxides and some anionic residues of biomolecules of interest for early regeneration [11]. However, calcium ions are fundamental in the coagulation process as well [12]. For the success of the implantation, it is crucial to achieve a rapid stabilization of the implant with the surrounding tissues. The clot or provisional matrix generates this preliminary stability. It consists of a three-dimensional fibrin network that contains platelets and growth factors. A fully functional clot orchestrates the attraction, housing, development, differentiation, and function of the cells in charge of the synthesis of the mature extracellular matrix [13]. Fibrin is an insoluble biopolymer formed from the progressive assembly of fibrinogen subunits. Thrombin allows the ends of fibrinopeptides to be separated from the main fibrinogen unit and the new ends to react with each other and form the three-dimensional fibrin network [14]. Developing this scaffold intimately with the implant surface opens the possibility, among others, of improving angiogenesis, reducing inflammation, and accelerating tissue repair directly on the surface. 

In this article, we set our objective to investigate the roles of the bioinorganic calcium ion in the apposition and formation of the fibrin clot at the implant surface and which may be the implications in the clinical integration of dental implants. To do this, a quartz crystal microbalance with dissipation (QCM-D) has been used to monitor the viscoelastic properties and settlement of the fibrin layer of surfaces modified with calcium ions (Ca) as compared to standard titanium surfaces (NoCa). The morphology of the resulting interfaces was characterized by Scanning Electron Microscopy (SEM). Regarding the early and long-term regeneration events, two clinical cases are presented in which these types of surfaces are employed.

## 2. Materials and Methods

Unless otherwise stated, all reagents were purchased from Scharlab S.L., Barcelona, Spain. Nanopure water used in this study was obtained by purification with a Diamond UV water system (Branstead International, Dubuque, IA, USA).

Blood from the patient (surgeries) or healthy volunteers (in vitro experiments) was collected into 0.4% (wt./vol.) sodium citrate-containing tubes (BTI Biotechnology Institute S.L., Vitoria, Spain) and used immediately or centrifuged following the manufacturer instructions to obtain platelet-rich plasma in accordance with the PRGF-Endoret^®^ Technology [4,15]. 

### 2.1. Quartz Crystal Microbalance with Dissipation Analysis (QCM-D)

**Figure 1:**
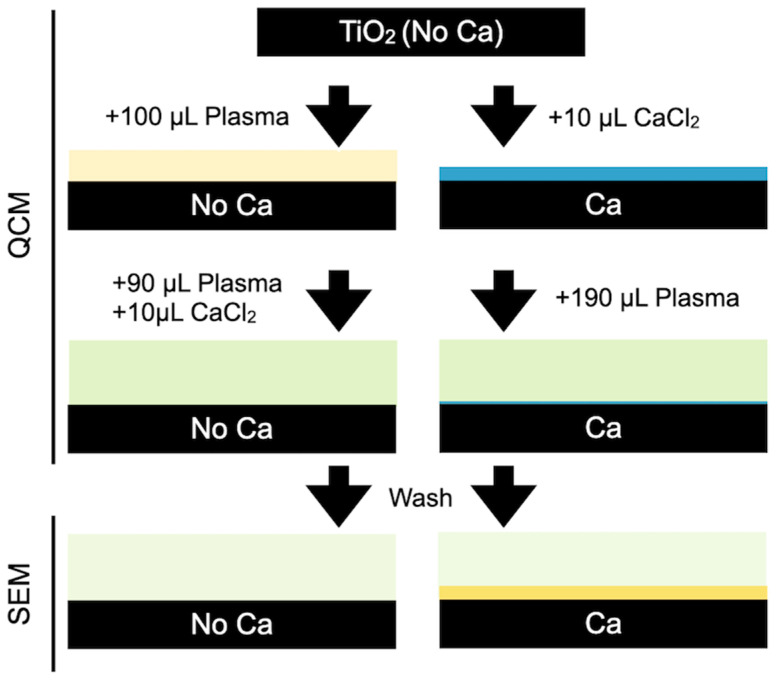
Scheme of the QCM experiments.

QCM-D experimental substrates were TiO_2_ sputtered SiO_2_ crystals with a 10 mm diameter on their working surface and gold-plated on the electrical contact surface (5 MHz Biolin Scientific, Gothenburg, Sweden). Immediately prior to each experiment, the surfaces were cleaned. First, the samples were sonicated in 2% sodium dodecyl sulfate solution (Sigma-Aldrich Chemie GmbH, Munich, Germany), filtered through a 0.2 mm pore diameter syringe filter (Millipore Sigma, Burlington, MA, USA), and rinsed under a stream of Nanopure water. The water was blown off with a filtered nitrogen stream. The dry surfaces were further treated with UV-Ozone for 30 min in a UV/Ozone cleaner (BioForce Nanosciences, Ames, IA, USA) that was pre-heated for 30 min immediately before use. The QCM-D measurements were performed on a Biolin Scientific Microbalance (Q-Sense). The TiO_2_-coated QCM-D sensor plates were installed into two parallel QCM-D liquid chambers connected with a temperature controller at 37 ± 0.1 °C. Figure 1 shows a scheme of the experiments. Briefly, 10 µL of 5 wt.% CaCl_2_ was injected into the left QCM-D chamber (Ca) and 100 µL of plasma into the right (NoCa). Both liquids covered completely the sensor plates and were left for around 30 min for stabilization and thermal equilibrium. Thereafter, 190 µL of plasma was injected into the right chamber (Ca) and a freshly prepared mixture of 10 µL CaCl_2_ 5 wt.% and 90 µL of plasma into the left chamber (NoCa). Both sensor plates contained the same final volume (200 µL) and had the same concentration of CaCl_2_ in the plasma (22.8 mM). During the QCM-D measurement, the frequency shift and dissipation change were simultaneously recorded at its first three overtones (*n* = 3, 5, 7). Recording was stopped after the frequency and dissipation signals were stabilized. The resulting interfaces were visually and microscopically analyzed (SEM). The experiments were performed in triplicates (*n* = 3 per surface type).

### 2.2. Blood Contact Experiments

For blood immersion experiments, commercial dental implants were employed. Ca-modified implants (BTI reference IIPUCA4010, unicCa^®^) and non-modified (BTI reference IIPU4010) followed the same proprietary process to convey roughness and cleanliness to the surfaces. Ca-modified samples were conditioned with 5% CaCl_2_ under clean room conditions prior to the sterilization of the implants (BTI). Freshly drawn blood from a young healthy patient in citrated tubes was poured into a borosilicate well. The implants were placed side by side and care was taken to avoid any contact with their surfaces at any point of the experiment. They were slowly introduced into the well leaving only the neck of the implants uncovered. After 20 min of incubation at room temperature, the implants were extracted, and their surfaces were analyzed visually and prepared for SEM examination. The experiment was repeated three times with identical results.

### 2.3. Scanning Electron Microscopy (SEM)

SEM images were taken with a Hitachi S-4800 (Hitachi High-Tech, Krefeld, Germany) at 15 kV acceleration voltage, around 10 mm working distance. After QCM and blood incubation experiments, respectively, QCM sensors and implants were immediately fixed for 1 h in 2 wt.% glutaraldehyde in 0.1 M sodium cacodylate buffer (pH = 7.4) at room temperature, washed 3 × 10 min with the 6.5 wt.% sucrose in the same cacodylate buffer, stained with 1 wt.% OsO_4_ in 0.1 M cacodylate buffer for 1 h at 4 °C in the dark, and finally washed 3 × 10 min with cacodylate buffer. The fixed samples were dehydrated in a series of solutions of increasing ethanol concentrations (30, 50, 70, 96, 3 × 100 vol.%). Each step took 10 min. The dehydrated samples were immersed in hexamethyldisilazane for 2 × 10 min and allowed to dry. The dry samples were coated with gold by sputtering for 180 s immediately before observation in an argon atmosphere in a JFC-1000 ion sputter (Jeol SAS, Croissy-sur-Seine, France) mounted on one of the ports of the SEM. Three images per implant (*n* = 9) at 1000× magnification were analyzed using the software ImageJ (version 1.53a; National Institutes of Health, Bethesda, MD, USA) to measure the Surface Coverage % (SC) of red blood cells, fibrin, and platelets on each surface. 

### 2.4. Implant Surgeries 

The patients subjected to implant surgeries were treated in the same private clinic by E.A. following the standard clinical practice. The first patient received implants due to severe lower jaw atrophy. The second patient was subjected to a maxillary sinus lift augmentation due to severe vertical bone resorption in the upper jaw. A more detailed description is provided later. A dental hygiene was performed prior to the surgery. For surgical planification, a computed axial tomography scan was used and analyzed by specialized software (BTI Scan^®^, BTI Biotechnology Institute, Vitoria, Spain). Demographic and clinical data were recorded in the patients’ clinical reports. Pre-surgical prophylaxis was administered (2 g of amoxicillin/600 mg of clindamycin and 1 g of acetaminophen). The amoxicillin treatment was continued during the next 5 days at a dose of 500 mg, three times a day. For pain control, acetaminophen 1 g/8 h or ibuprofen 600 mg/8 h were prescribed. After infiltrative anesthesia, a full-thickness flap was elevated, and the implant sites were prepared using a low-speed 125 rpm drilling procedure without irrigation [16]. The preparation of blood plasma (PRGF) obtained with the Endoret^®^ technology was performed following the manufacturer’s instructions [4,15]. Before implant insertion, the implant sites were filled with PRGF-Endoret^®^, fraction 2. In both cases, two 5.5 mm diameter and 5.5 mm long titanium implants with their surface modified with calcium ions (unicCa^®^, BTI Biotechnology Institute) were used. The insertion speed was 30 rpm. Finally, a panoramic radiograph was taken just after the intervention to verify the adequate placement of the implants.

For the post-surgical clinical assessment, patients attended the private clinic periodically, first after the surgical phase (10 days, 1, 3, 6 months, and 1 year) and then on a yearly basis, performing panoramic radiographs at each visit. Measurements on the panoramic radiographs were performed by computer software (Sidexis XG, Sirona Dental Systems, Bensheim, Germany). The radiographs were calibrated by the known implant length.

### 2.5. Data Analysis 

The data are shown as means ± Standard Deviation. We confirmed data normality prior to comparisons (Shapiro–Wilk). We determined the differences between the means by a two-sample independent Student’s two-tailed homoscedastic *t*-test between surfaces. We considered statistical significance for *p* < 0.01. The software used for statistical analysis was Origin v7.5 (OriginLab Corporation, Northampton, MA, USA).

## 3. Results

### 3.1. Quartz Crystal Microbalance with Dissipation (QCM-D) Experiments

Quartz crystal microbalance records changes in the resonance frequency (*f*) of a piezoelectric element (quartz crystal) due to the pressure exerted by a given amount of adsorbed mass. The way in which the crystal dissipates energy gives an index of the changes that occur in the adsorbed layer: the viscosity and the hardness of the adsorbed biofilm, among others.

**Figure 2:**
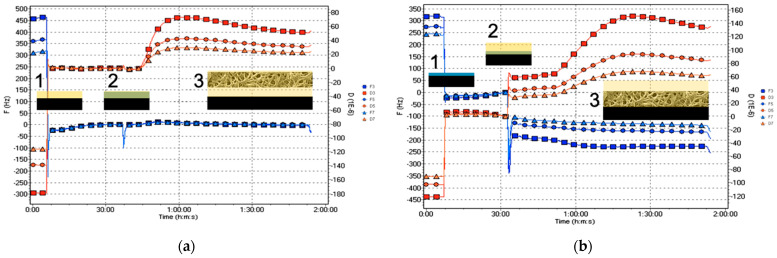
Evolution of the third, fifth, and seventh overtones of frequency (F, blue) and dissipation (D, red) of the samples (**a**) NoCa (Plasma first) and (**b**) Ca (Ca first) according to the different modifications described in Figure 1: the frequency and dissipation changes at points 1–3 are shown in Table 1. #1 Surfaces (a) unmodified (NoCa), or (b) modified with calcium ions (Ca) prior to any subsequent addition (contact with air). #2 Plasma deposition (without Ca in b). #3 Coagulation.

Figure 2 shows the frequency and dissipation evolution as a function of time. The reduction in the initial resonance frequency shows that both surfaces passed from air contact to liquid contact: 100 μL plasma for the NoCa samples (Figure 2a #1) and 10 μL CaCl_2_ for the Ca samples (Figure 2b #1). Once the crystal is stabilized, the volumes and amounts of calcium are equalized. To reach 200 μL in both cells, a mixture of 10 μL of CaCl_2_ and 90 μL of plasma is added to the NoCa QCM cell (Figure 2a #2) and 190 μL of plasma to the Ca cell (Figure 2b #2). In this way, the effects on the clot formation of calcium ions at the same surfaces can be analyzed depending on whether the ions were previously at the surfaces (Ca) or present in the circulating milieu (NoCa).

Figure 2b #2 shows the decrease in the resonance frequency due to the addition of plasma to the Ca cell, ∆*f* = −40 Hz, which indicates surface adsorption. Increased dissipation and the separation of the overtones (red lines) indicate, in turn, the formation of the clot: D = (35 ± 4) × 10^−6^. This phenomenon also occurs at the surface not previously exposed to calcium D = (35 ± 3) × 10^−6^. However, in this case, there is no variation of the resonance frequency, i.e., ∆*f* = 0 (Figure 2a #2), meaning there is no adsorption of the clot on the surface. Table 1 and Table 2 summarize the resonance frequency (*f*) and dissipation (D) of the NoCa and Ca systems, respectively.

**Figure 3:**
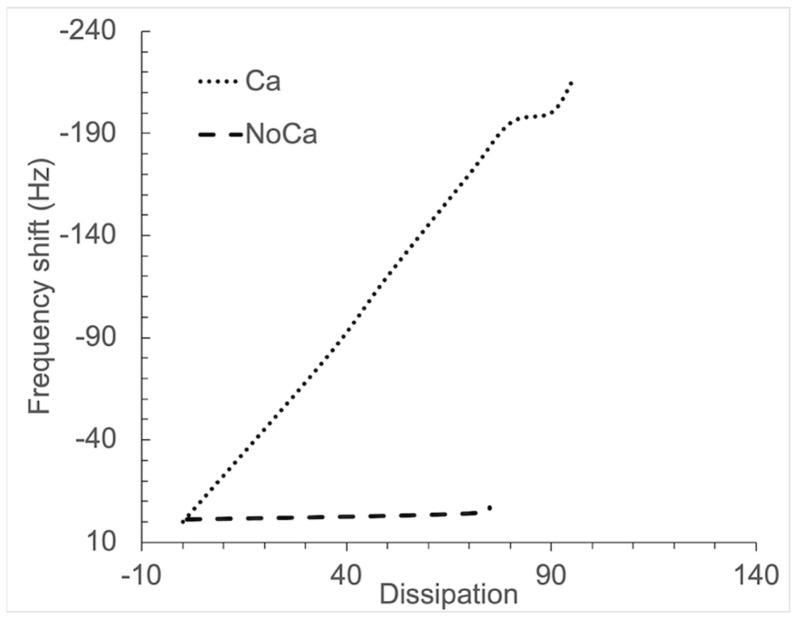
Representative *f*/D plots of the surfaces. The frequency shift (*f*) is given on the Y-axis, and registration starts for all surfaces when Ca^2+^ is added (*f* = 0). The corresponding dissipation values are given on the X-axis. The experimental time span is 45 min.

Figure 3 shows a representative *f*/D plot of the surfaces. The *f*/D relationship is an indication of the viscoelasticity due to coagulation. A low value of dissipation means higher rigidity. The *f*/D ratio can be thus used as an estimation of the clot density. A high *f* combined with a low D value would indicate a high-density clot. The Ca plot appears to illustrate the formation of a high-density clot while the NoCa plot would correspond to a low-density clot. However, NoCa *f* values close to 0 may be rather showing that the clot is not firmly attached to the surface and slips over the surface, leading to an underestimation of mass. 

**Figure 4:**
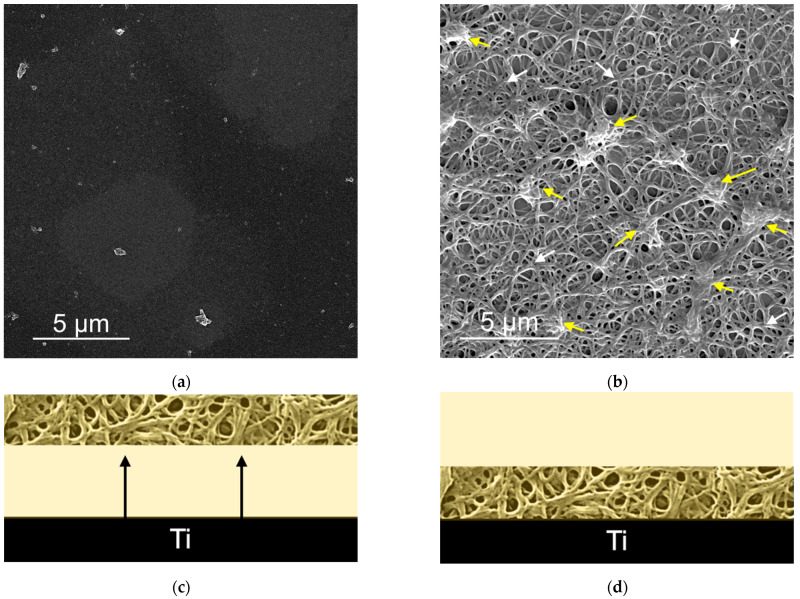
Scanning Electron Micrographs of the QCM sensor surfaces after the QCM experiments. (**a**) NoCa surfaces showed no adsorbed material. (**b**) Ca surfaces had a fibrin film (white arrows) with embedded platelets and platelet aggregates (yellow arrows). Below each micrograph, a scheme illustrating the phenomena at (**c**) NoCa and (**d**) Ca is proposed. The black arrows in (**c**) indicate detachment of the clot from the surface. At (**d**), the clot remains attached to the surface.

After the QCM measurements, the sensors were dismounted from the QCM cells and prepared for SEM examination. At that point, it became macroscopically clear that the clot had been bound to the surfaces pre-exposed to Ca ions, but not to the surfaces in which there were no Ca ions initially, even though the clot was also visible and floating (images not shown). The SEM images confirmed the macroscopic observation (Figure 4).

### 3.2. Blood Contact Experiments

**Figure 5:**
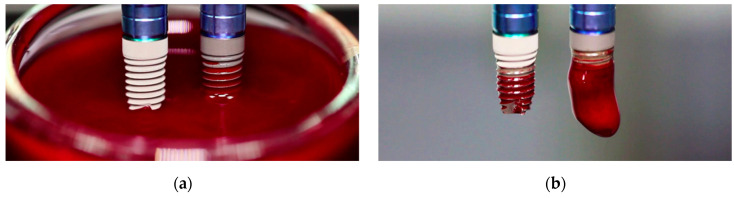
Implant immersion experiments in blood. (**a**) First contact of the implant surfaces with blood. (**b**) Resulting surfaces after 20 min incubation at room temperature in the blood-containing well.

We immersed simultaneously the two implants with the two different surfaces studied in a well containing citrated blood from a healthy donor for 20 min. Figure 5 shows the two implants: an implant with a standard titanium surface (NoCa, left) and an implant modified with calcium ions (Ca, right). The implants were simultaneously put in contact with blood first and immersed then into the blood well. For the implants modified with Ca ions, blood rapidly covered the implant surface by capillarity. For the NoCa implants, though, blood progressively met the implant surface as the implant was introduced into the blood well (Figure 5a). After 20 min of incubation at room temperature, the implants were simultaneously pulled out of the blood. The implants previously modified with Ca clearly showed a clot formed around them while the implants without Ca did not (Figure 5b).

**Figure 6:**
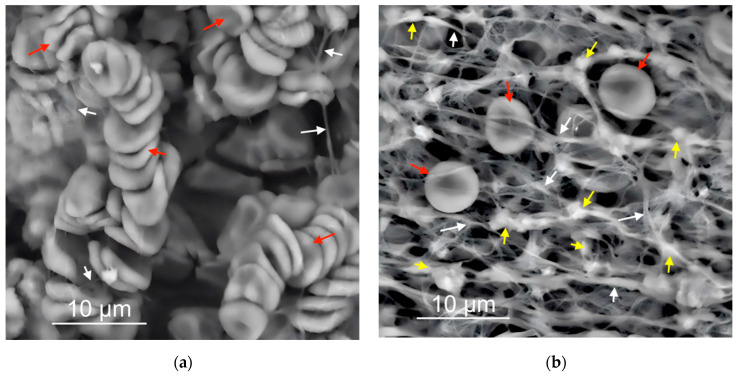

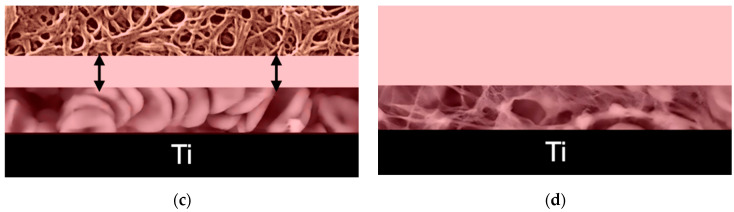
(**a**,**b**) Scanning Electron Micrographs of the implant surfaces after blood immersion experiments. NoCa surfaces (**a**) showed a thick layer mainly composed of red blood cells (red arrows) with scarce fibrillar material (white arrows) while Ca surfaces (**b**) showed a dense fibrin network (white arrows) with embedded platelets (yellow arrows) and some interspersed red blood cells (red arrows). Below each micrograph, a scheme illustrating the phenomena is proposed (**c**,**d**). The black arrows in (**c**) indicate that most of the fibrin network stays away from NoCa surfaces. Only a thick layer mostly composed of red blood cells stays attached to the NoCa surfaces. On the Ca surfaces (**d**), the clot forms at the surface and contains red blood cells and platelets entrapped within the surface-bound fibrin.

Electron microscopy images of the morphology of the surfaces resulting from these tests are shown in Figure 4a,b and Figure 6a,b. The images are representative of the three samples per surface type used in each experiment. The vertical distribution of these structures is also schematized in Figure 4c,d and Figure 6c,d. After the blood plasma experiments, we found no distinguishable plasma biomolecules at the NoCa surface (Figure 4a) while at the Ca surface (Figure 4b) we found 95 ± 4% Surface Coverage by fibrin (white arrows) and SC = 25 ± 15% of fibrin-embedded platelets and platelet aggregates (yellow arrows).

After the blood immersion experiments (Figure 6a,b), we found that on the NoCa surfaces, red blood cells yielded an SC = 67 ± 12% while on the Ca surfaces, only SC = 9 ± 3% (red arrows). Fibrin resulted in an SC = 3 ± 2% on the NoCa surfaces and 93 ± 5% on the Ca surfaces (white arrows). Platelets were not visible on the NoCa surfaces but they were present on the Ca surfaces embedded within the fibrin network (SC: 40 ± 20%, yellow arrows). All differences between the surfaces were statistically significant (*p* < 0.01). 

In summary, we found that, in the case of the samples previously modified with Ca, the provisional matrix is formed right at the surface and is mainly composed of fibrin and platelets (Figure 4b). In the case of exposure to blood, we also see red blood cells scattered in that same network (Figure 6b). However, if there is no such previous modification with Ca, the surfaces exposed to the plasma do not show the adsorption of any three-dimensional network or provisional matrix (Figure 4a), and, in the case of exposure to blood, a thick coating with red blood cells is obtained with hardly any visible remains of the fibrin network or other elements of the provisional matrix (Figure 6a). 

### 3.3. Clinical Cases

We have evaluated two clinical cases in which the characteristics of the Ca-ion unicCa^®^ surface in terms of initial regeneration and blood clot formation may be particularly beneficial. Both were challenging cases that followed a minimally invasive approach.

#### 3.3.1. Vertical Bone Atrophy in the Mandible

**Figure 7:**
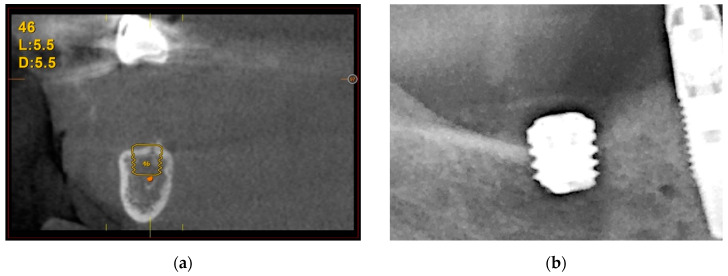

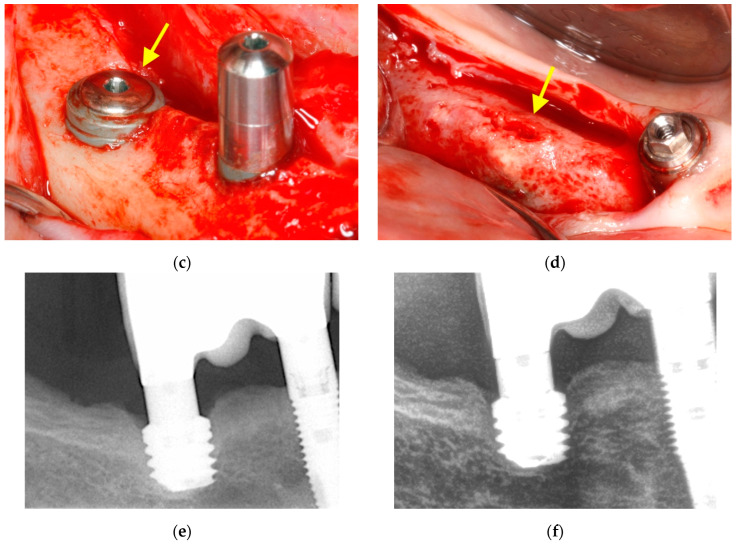
Vertical bone atrophy in the jaw with a residual bone height of 4 mm (**a**). Insertion of the dental implant leaving a part of the implant exposed, which was covered using bone recovered from the site preparation mixed with PRGF-Endoret^®^ (**b**,**c**). At 4 months (**d**,**e**), vertical bone regeneration can be observed covering the implant (yellow arrow). Stability of the regenerated bone at 5 years follow-up (**f**).

The first case is shown in Figure 7 and corresponds to vertical bone atrophy in the lower jaw with a residual bone height of 4 mm above the alveolar nerve (Figure 7a). The placement of the implant was performed just above the nerve leaving a portion of the implant neck exposed buccolingually (Figure 7b). The exposed area of the implant was covered using the patient’s own bone mixed with PRGF-Endoret^®^ (Figure 7c). The slow-drilling procedure for the implant-site preparation (125 rpm without irrigation) allows the recovery of bone from the patient that, once mixed with PRGF-Endoret^®^, forms a flexible membrane with embedded bone particles. This membrane can be shaped to fill biological gaps or to overcorrect situations like the one described above [17]. Figure 7d,e shows how already, 4 months after the placement of the implant, the regenerated bone is covering the implant completely. The stability of this bone is maintained 5 years after the surgery (Figure 7e). 

#### 3.3.2. Maxillary Sinus Floor Augmentation

**Figure 8:**
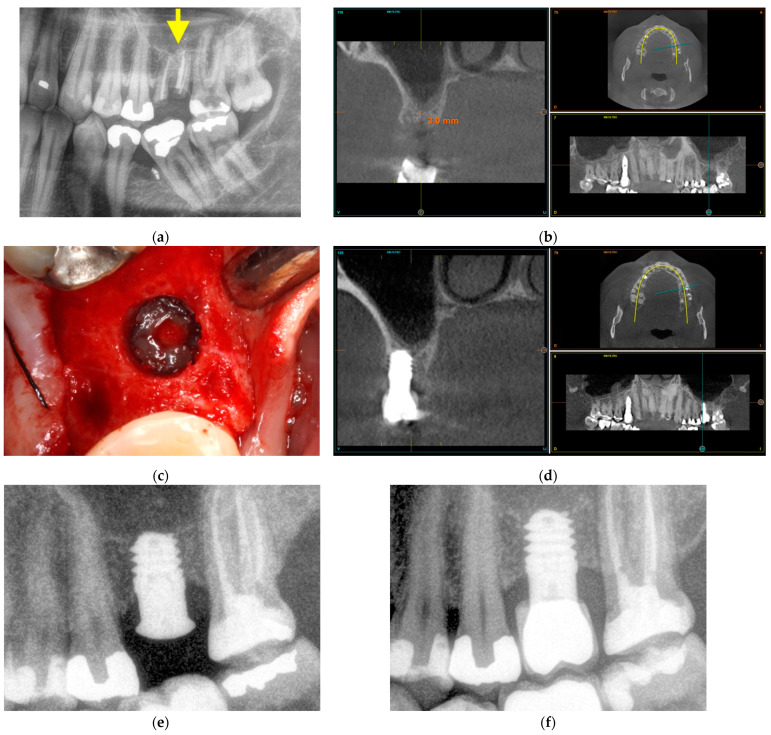
Maxillary sinus floor augmentation. Prior extraction of the infected tooth roots, marked with a yellow arrow (**a**). Three months after extraction, the residual bone height is 3 mm (**b**). The sinus membrane was gently pushed upwards with autologous bone recovered from the site preparation mixed with PRGF-Endoret^®^ (**c**). A 5.5 mm short implant is placed at the implant site to support the sinus floor elevation apically (**d**). Three months after implant placement, the bone height around the implant was 4.5 mm (**e**). Five years after the placement of a short implant, the measured bone height at the implant site was 7.5 mm (**f**).

The second case corresponds to an upper jaw with severe vertical bone resorption (Figure 8). The tooth roots were removed due to infection (Figure 8a), and the site was filled with PRGF-Endoret^®^. After three months, the available vertical height was 3 mm (Figure 8b). In the surgery plan, the bone density of the site was measured, and an extra short unicCa^®^ implant 5.5 mm in length was planned. The surgery was carried out with a frontal attack drill (Figure 8c) up to the sinus membrane. The membrane is lifted gently with autologous bone from the osteotomy mixed with PRGF-Endoret^®^ and retained in position after the positioning of the implant (Figure 8d,e). Soon after the surgery, the peri-implant bone height was 4.5 mm. Five years after the surgery, the bone height was stable around the implant and in its apical part (Figure 8f). The new vertical bone height was 7.5 mm. 

## 4. Discussion

In this work, we have evaluated the effects of exposing titanium to calcium ions prior to their contact with human blood and blood plasma. We have monitored the process of blood and blood plasma coagulation on these surfaces as compared to regular unmodified titanium implant surfaces. The QCM-D technique used permits evaluating the changes that occur at the adsorbed layer. The adsorbed mass and the thickness, viscosity, and hardness of the films formed are common outputs of this technique. The Sauerbrey equation relates the change of the resonance frequency (*f*) proportionally to the added mass (m) [18]. However, in the case of viscoelastic films, where the dissipation to frequency shifts ratio (D/*f*) is typically below 0.2 × 10^−6^ Hz^−1^, the Sauerbrey equation is no longer considered a good approximation [19]. Therefore, the mass and thickness of the deposited films could not be calculated. Looking into the future, the application of the Voigt model could allow us to obtain numerical data of the biofilms. The Voigt model has been already used in the past for the determination of the adsorbed mass and thickness of viscoelastic biofilms using a complex shear modulus. The storage modulus is the real part, and the loss modulus is the imaginary one [19,20,21]. 

However, the qualitative data obtained are enough for the purpose of this work: on surfaces previously modified with calcium, coagulation occurs within the adsorbate at the surface. In contrast, when the calcium ions are not previously present at the surfaces but are presented together with the blood plasma solution, coagulation occurs, but the fibrin network does not stay attached to the surface. This observation agrees with previous research showing that at unmodified Ti surfaces, the fibrin clot slips over and does not attach to the surfaces [22]. The macroscopic aspect of the surfaces obtained after the experiments in blood and blood plasma supports this observation, as well as the surface coverage measurements from the electron micrographs of the surfaces. Moreover, the first interaction of the implant surfaces with blood showed their respective wetting behavior. In contact angle experiments, typically, the frontier between hydrophilic (wetting) and hydrophobic (non-wetting) surfaces is set at an angle between water and the surface of 90° [23]. Wettability studies at Ca-enriched surfaces have shown that this modification on titanium renders the surfaces completely hydrophilic or superhydrophilic (contact angles below 5°), while the control surface is only slightly hydrophilic (contact angles above 65°) [10,12]. Superhydrophilicity allows the implant to render its surface immediately available for the upcoming regenerative events. This is especially relevant in the case of small-sized implants with less available surface. 

On the other hand, the formation of the provisional matrix directly on the surface of the implant can have advantageous effects on the generation of the definitive extracellular matrix as an early support for implant stability. The increased levels of platelet activation and clot formation were associated with higher levels of osseointegration in the femoral condyle of rabbits at 2 and 8 weeks post-implantation [12]. Early healing also increased in surfaces with Ca-ions in the early periods of implantation in the dog mandible with respect to other hydrophilic surfaces [24]. The effects of calcium ions in the protection of the native titanium oxide pureness were related to an increased osteoblast cell function and bone regeneration in a goat tibia model 3 months after implantation with respect to non-modified standard implants [25]. Ionic calcium seems to be the key leading to the stimulation of the early healing events. Compared to surfaces with calcium in a non-ionic form as a part of a nanohydroxyapatite coating (DCD or Discrete Crystalline Deposition surfaces), Ca-ion unicCa^®^ surfaces resulted in significantly increased levels of new bone apposition, especially at 2 and 4 weeks post-implantation in the dog mandible [26]. The effects of the protein adsorbate were recently investigated with respect to regular titanium implant surfaces. Ca-ion unicCa^®^ surfaces boost coagulation using the common pathway rather than the intrinsic or contact activation pathway as detected by the coagulation factors in these surfaces. These results were correlated with significantly higher levels of osseointegration in the rabbit model [5]. In another study focusing on the reactivity of implants to peri-implant disease formation and progression in a dog model with ligatures and no plaque control, the marginal bone loss at Ca-ion unicCa^®^ surfaces was 33% lower than implants stored in NaCl solution (SLActive^®^) [27]. The results of this in vivo study are in accordance with previous in vitro experiments in which Ca-ion unicCa^®^ surfaces showed significantly less bacterial adhesion and better mammalian cell functions than unmodified controls in the presence and absence of natural saliva and blood plasma [10]. 

In a recent clinical study with patients displaying systemic diseases of a different nature showed that rehabilitation with calcium ion-modified unicCa^®^ implants associated with plasma rich in growth factors proved to be a safe and effective treatment with results comparable to those found in healthy patients after 5 years [3].

Another clinical study compared Ca-ion unicCa^®^ dental implants and the same implants without calcium ions. The clinical model was vertical bone atrophy in the posterior sector of the upper jaw. The implants were placed with the transalveolar sinus lift technique using the low-rev milling technique and the application of PRGF-Endoret^®^. The results of this research indicate that modifying implants with calcium ions significantly improves bone stability around the dental implant and reduces its failure rate. This reduction was related to the improvement of osseointegration mediated by the release of calcium ions from the surface of the dental implant [28].

In the two clinical cases here presented, we found vertical bone regeneration, coronally and apically, respectively. The effects of calcium ions on unicCa^®^ surfaces allowed the use of minimally invasive techniques in challenging situations with severe vertical bone defect. Some weeks after the surgeries, the implants succeed in stimulating vertical bone growth and the thickening of the surrounding supporting bone, which is maintained throughout the following years. The use of the Ca-ion modified unicCa^®^ implant surface represents a valuable tool in short or narrow implants to render all available implant surface functional in the quest for a fast implant stabilization in these challenging situations.

## 5. Conclusions

In this study, we have evaluated the mechanistic properties of dental implant surfaces modified with calcium ions on the promotion of the provisional matrix formation directly on their surface. The calcium ion, present in numerous key steps of the bone regeneration process, has been capable of differentially stimulating the biopolymerization of fibrin and the platelet activation directly on the implant surfaces. The unmodified surfaces were not initially capable of fixating the provisional matrix to the surface, suggesting that in the presence of standard implant surfaces, the initial events of regeneration occur in the microenvironment of the implant surface but are not intimately linked to it. The adsorption and differential formation of the provisional matrix on the Ca ion surface has a significant impact on the osseointegration of the implants, especially in the very delicate initial period of healing. The clinical importance of this surface modification that improves the osseointegration of the implants lies in the fact that it can allow to address more complex cases, reduce healing times, and reduce the rates of early failure. However, more research is needed to fully unwrap the link between the early regeneration phases around implants and long-term implant osseointegration.

## Figures and Tables

**Table 1:** Resonance frequency (*f*) and dissipation (D) of the fifth overtone due to changes in the adsorption on the NoCa surface. Step 1 corresponds to the passage from air to plasma (liquid). Step 2 corresponds to the addition of plasma with CaCl_2_ (liquid) to the plasma (liquid). Step 3 corresponds to coagulation.

Steps	Volume (µL)	*f* (Hz)	D (×10^−6^)
1. Plasma	100	−360 ± 15	−175 ± 13
2. Plasma + CaCl_2_	100	0	0
3. Coagulation	200	0	35 ± 3

**Table 2:** Resonance frequency (*f*) and dissipation (D) of the fifth overtone due to changes in the adsorption on the Ca surface. Step 1 corresponds to the passage from air to CaCl_2_ (liquid). Step 2 corresponds to the addition of plasma (liquid) to the CaCl_2_ (liquid). Step 3 corresponds to coagulation.

Steps	Volume (µL)	*f* (Hz)	D (×10^−6^)
1. CaCl_2_	10	275 ± 15	−380 ± 21
2. Plasma	190	−125 ± 12	0
3. Coagulation	200	−165 ± 17	35 ± 4

## Data Availability

Additional data supporting the reported results are available upon request.

## References

[1] Esposito M., Hirsch J.M., Lekholm U., Thomsen P. (1998). Biological Factors Contributing to Failures of Osseointegrated Oral Implants. (I). Success Criteria and Epidemiology. Eur. J. Oral Sci..

[2] Tejero R., Anitua E., Orive G. (2014). Toward the Biomimetic Implant Surface: Biopolymers on Titanium-Based Implants for Bone Regeneration. Prog. Polym. Sci..

[3] Mozzati M., Gallesio G., Menicucci G., Manzella C., Tumedei M., Del Fabbro M. (2021). Dental Implants with a Calcium Ions-Modified Surface and Platelet Concentrates for the Rehabilitation of Medically Com-Promised Patients: A Retrospective Study with 5-Year Follow-Up. Materials.

[4] Anitua E., Montalvillo A., Eguia A., Alkhraisat M.H. (2021). Clinical Outcomes of Dental Implants Placed in the Same Region Where Previous Implants Failed Due to Peri-Implantitis: A Retrospective Study. Int. J. Implant Dent..

[5] Anitua E., Cerqueira A., Romero-Gavilán F., García-Arnáez I., Martinez-Ramos C., Ozturan S., Azkargorta M., Elortza F., Gurruchaga M., Goñi I. (2021). Influence of Calcium Ion-Modified Implant Surfaces in Protein Adsorption and Implant Integration. Int. J. Implant Dent..

[6] Romero-Gavilán F., Cerqueira A., Anitua E., Tejero R., García-Arnáez I., Martinez-Ramos C., Ozturan S., Izquierdo R., Azkargorta M., Elortza F. (2021). Protein Adsorption/Desorption Dynamics on Ca-Enriched Titanium Surfaces: Biological Implications. J. Biol. Inorg. Chem..

[7] Othman Z., Cillero Pastor B., van Rijt S., Habibovic P. (2018). Understanding Interactions between Biomaterials and Biological Systems Using Proteomics. Biomaterials.

[8] Tejero R., Rossbach P., Keller B., Anitua E., Reviakine I. (2013). Time-of-Flight Secondary Ion Mass Spectrometry with Principal Component Analysis of Titania-Blood Plasma Interfaces. Langmuir.

[9] Anitua E., Flores C., Flores J., Alkhraisat M.H. (2019). Clinical Effectiveness of 6.5-Mm-Long Implants to Support Two-Implant Fixed Prostheses in Premolar-Molar Region: The Influence of Immediate Loading and the Length of Splinting Implant. J. Prosthodont..

[10] Anitua E., Tejero R., Pacha-Olivenza M.Á., Fernández-Calderón M.C., Delgado-Rastrollo M., Zalduendo M.M., Troya M., Pérez-Giraldo C., González-Martín M.L. (2018). Balancing Microbial and Mammalian Cell Functions on Calcium Ion-Modified Implant Surfaces. J. Biomed. Mater. Res.—Part B Appl. Biomater..

[11] Ellingsen J.E. (1991). A Study on the Mechanism of Protein Adsorption to TiO_2_. Biomaterials.

[12] Anitua E., Prado R., Orive G., Tejero R. (2014). Effects of Calcium-Modified Titanium Implant Surfaces on Platelet Activation, Clot Formation, and Osseointegration. J. Biomed. Mater. Res.—Part A.

[13] Anitua E., Tejero R., Zalduendo M.M., Orive G. (2012). Plasma Rich in Growth Factors (PRGF-Endoret) Promotes Bone Tissue Regeneration by Stimulating Proliferation, Migration and Autocrine Secretion on Primary Human Osteoblasts. J. Periodontol..

[14] Evans-Nguyen K.M., Fuierer R.R., Fitchett B.D., Tolles L.R., Conboy J.C., Schoenfisch M.H. (2006). Changes in Adsorbed Fibrinogen upon Conversion to Fibrin. Langmuir.

[15] Anitua E. (1999). Plasma Rich in Growth Factors: Preliminary Results of Use in the Preparation of Future Sites for Implants. Int. J. Oral Maxillofac. Implants.

[16] Anitua E., Murias-Freijo A., Alkhraisat M.H., Orive G. (2015). Implant-Guided Vertical Bone Augmentation around Extra-Short Implants for the Management of Severe Bone Atrophy. J. Oral Implantol..

[17] Anitua E., Carda C., Andía I. (2007). A Novel Drilling Procedure and Subsequent Bone Autograft Preparation: A Technical Note. Int. J. Oral Maxillofac. Implants.

[18] Sauerbrey G. (1959). Verwendung von Schwingquarzen Zur Wägung Dünner Schichten Und Zur Mikrowägung. Z. Phys..

[19] Voinova M.V., Rodahl M., Jonson M., Kasemo B. (1999). Viscoelastic Acoustic Response of Layered Polymer Films at Fluid-Solid Interfaces: Continuum Mechanics Approach. Phys. Scr..

[20] Höök F., Kasemo B., Nylander T., Fant C., Sott K., Elwing H. (2001). Variations in Coupled Water, Viscoelastic Properties, and Film Thickness of a Mefp-1 Protein Film during Adsorption and Cross-Linking: A Quartz Crystal Microbalance with Dissipation Monitoring, Ellipsometry, and Surface Plasmon Resonance Study. Anal. Chem..

[21] Irwin E.F., Ho J.E., Kane S.R., Healy K.E. (2005). Analysis of Interpenetrating Polymer Networks via Quartz Crystal Microbalance with Dissipation Monitoring. Langmuir.

[22] Andersson M., Andersson J., Sellborn A., Berglin M., Nilsson B., Elwing H. (2005). Quartz Crystal Microbalance-with Dissipation Monitoring (QCM-D) for Real Time Measurements of Blood Coagulation Density and Immune Complement Activation on Artificial Surfaces. Biosens. Bioelectron..

[23] Law K.Y. (2014). Definitions for Hydrophilicity, Hydrophobicity, and Superhydrophobicity: Getting the Basics Right. J. Phys. Chem. Lett..

[24] Favero R., Lang N.P., Salata L.A., Neto E.C.M., Caroprese M., Botticelli D. (2016). Sequential Healing Events of Osseointegration at UnicCa^®^ and SLActive^®^ Implant Surfaces: An Experimental Study in the Dog. Clin. Oral Implants Res..

[25] Anitua E., Piñas L., Murias A., Prado R., Tejero R. (2015). Effects of Calcium Ions on Titanium Surfaces for Bone Regeneration. Colloids Surfaces B Biointerfaces.

[26] Favero R., Botticelli D., Antunes A.A., Martinez Sanchez R., Caroprese M., Salata L.A. (2016). Sequential Healing at Calcium- versus Calcium Phosphate-Modified Titanium Implant Surfaces: An Experimental Study in Dogs. Clin. Implant Dent. Relat. Res..

[27] Favero G., Apaza Alccayhuaman K.A., Silva E.R., Bengazi F., Urbizo J., Kotsu M., Botticelli D. (2020). Effect of Lack of Plaque Control after the Surgical Treatment of Peri-Implantitis at Surfaces with Different Characteristics: An Experimental Study in Dogs. Oral Maxillofac. Surg..

[28] Anitua E., Piñas L., Alkhraisat M.H. (2017). Early Marginal Bone Stability of Dental Implants Placed in a Transalveolarly Augmented Maxillary Sinus: A Controlled Retrospective Study of Surface Modification with Calcium Ions. Int. J. Implant Dent..

